# Threatenings of Disease

**Published:** 1856-12

**Authors:** 


### THREATENINGS OF DISEASE.

All serious diseases give their far-off warnings. Intelligence and careful observation would make a doctor's calling almost a sinecure. A gradual failure of the memory is a sure indication of approaching bodily infirmity or decay. Another important fact is, if any set of muscles are unduly exercised, they will lose their power; so also, if any function of the mind or brain is unduly stimulated, the result is temporary prostration or permanent destruction, according to the intensity and duration of that stimulus. Thus it is, that the young, who learn by memory, if highly stimulated to learn, become precocious, and either die early, or disappoint the expectations of their friends by settling down into mortifying mediocrity. Hence

1. Let the young learn slowly.
2. Under intense bodily or mental application, if you find your memory failing you, as you value bodily health, and the mind itself, break away at once from all your engagements, and spend weeks together in out-door recreations.

